# The impact of dyslipidemia on prognosis of patients after endovascular abdominal aortic aneurysm repair

**DOI:** 10.3389/fcvm.2024.1341663

**Published:** 2024-03-25

**Authors:** Xin Luo, Qiang Guo, Jiarong Wang, Yiyuan Li, Jichun Zhao, Bin Huang, Xiyang Chen

**Affiliations:** Department of Vascular Surgery, West China Hospital of Sichuan University, Chengdu, Sichuan, China

**Keywords:** abdominal aortic aneurysm, dyslipidemia, endovascular aneurysm repair, type I endoleak, follow-up outcome

## Abstract

**Introduction:**

Dyslipidemia is common in patients with abdominal aortic aneurysm (AAA). However, there is insufficient research on the impact of dyslipidemia on the postoperative outcomes of patients with AAA after endovascular aortic aneurysm repair (EVAR). This study aimed to determine the impact of dyslipidemia on the prognosis of patients with AAA treated with EVAR.

**Method:**

We retrospectively reviewed patients with AAA who underwent EVAR at our hospital between 2010 and 2020. The baseline characteristics and prognoses of patients in the dyslipidemia and non-dyslipidemia groups were analyzed.

**Results:**

A total of 641 patients were included; the prevalence of dyslipidemia in patients with AAA was 42.3% (271/641), and the mean follow-up time was 63.37 ± 26.49 months. The prevalence of diabetes (10.0% vs. 15.1%, *P* = 0.050), peripheral arterial disease (17.3% vs. 25.8%, *P* = 0.018), and chronic kidney disease (3.0% vs. 6.3%, *P* = 0.043) was higher in the dyslipidemia group. The three-year all-cause mortality rate after EVAR was 9.98% (64/641), and there was no difference in the incidence of all-cause mortality (10.27% vs. 9.59%, *P* = 0.778) between the two groups. A total of 36 (5.62%) major adverse cardiovascular and cerebrovascular events (MACCEs) were observed within 3 years and were more common in patients with dyslipidemia (2.97% vs. 9.59%, *P* < 0.001). The incidence of stent-related complications in all patients was 19.97% (128/641), and there was no difference in the incidence of stent-related complications between the two groups (22.16% vs. 16.97%, *P* = 0.105); however, the incidence of type I endoleak in the dyslipidemia group was lower than that in the non-dyslipidemia group (9.19% vs. 4.06%, *P* = 0.012). Cox-regression analysis showed that high level of high-density lipoprotein cholesterol (HDL-C) was the protective factor (HR, 0.203, 95% CI, 0.067–0.616, *P* = 0.005) for MACCES, but it was the risk factor for type I endoleak (HR, 2.317, 95% CI, 1.202–4.466, *P* = 0.012).

**Conclusion:**

Dyslipidemia did not affect the mortality of patients with AAA who underwent EVAR; however, it may increase the incidence of MACCEs. Dyslipidemia may decrease the incidence of type I endoleaks after EVAR; however, further studies are warranted. We should strengthen the postoperative management of patients with dyslipidemia, prevent the occurrence of MACCEs.

## Background

Abdominal aortic aneurysm (AAA) refers to the dilation of the abdominal aorta with a diameter greater than 3 cm (1, 2). It is more common in male smokers over the age of 65 years and other risk factors include dyslipidemia, hypertension, and other common risk factors for cardiovascular diseases (3, 4). Endovascular aortic aneurysm repair (EVAR) has become the main treatment for AAA due to its minimally invasive nature, excellent short-term and acceptable long-term prognosis (5). Endoleak is a common complication after EVAR, which refers to the continuous blood flow in the aneurysm sac after EVAR. Type I endoleak refers to incomplete sealing of the proximal aortic attachment site (IA) or distal iliac artery attachment site (IB). Type II endoleak is defined as the continuous filling of the aneurysm sac by the patent lumbar artery or inferior mesenteric artery, type III endoleak occurs when there is incomplete seal between components or component separation (6–8).

Dyslipidemia is common in patients with AAA. A previous study has shown that total cholesterol (TC) and low-density lipoprotein cholesterol (LDL-C) play a role in the pathogenesis of AAA (9). Patients with small-diameter AAA may benefit from treatment that reduces LDL-C (10). High-density lipoprotein (HDL) reduction and dysfunction are also related to AAA formation (11). Dyslipidemia also affects the surgical treatment of AAA. A study pointed out that type II endoleak after EVAR for AAA is associated with dyslipidemia (12). However, there is still insufficient research on the impact of dyslipidemia on the postoperative outcomes of patients with AAA after EVAR.

Therefore, we conducted a retrospective study to screen patients with AAA with concomitant dyslipidemia and explore the impact of dyslipidemia on EVAR.

## Methods

### Patients

**Figure 1:**
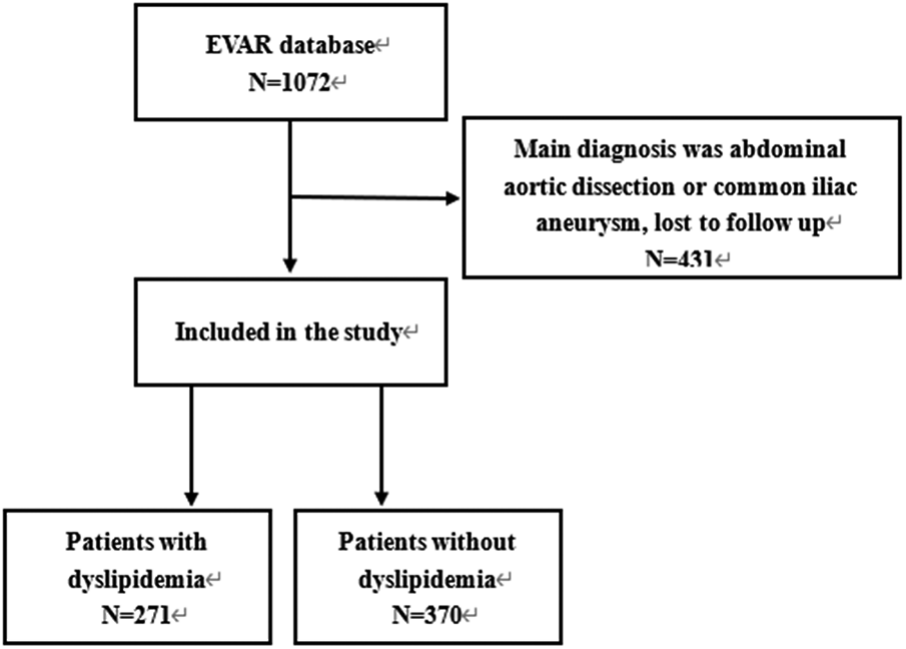
Patients inclusion and exclusion.

The medical records of 1,072 patients who received EVAR between 2010 and 2020 in our hospital were reviewed, and all patients with AAA who underwent EVAR were included, except for cases with: (1) The follow-up time less than three years or follow-up was lost; (2) The main diagnosis being abdominal aortic dissection or common iliac artery aneurysm. Finally, as shown in the Figure 1, 641 patients with AAA were included, and all patients were followed up by outpatient clinics or telephone for at least 3 years. The patients were divided into dyslipidemia and non-dyslipidemia groups based on whether they had dyslipidemia before EVAR. This study was approved by the ethics committee of West China Hospital, Sichuan University, the approve number was 20221827. The requirement for informed consent was waived due to de-identified patient information.

### Data collection

We collected the anatomical parameters of AAA, including neck angle, neck length, and maximum diameter, by comparing contrast-enhanced computed tomography and angiography. We collected the surgical records and baseline characteristics of each patient through an electronic medical record system, including age, sex, underlying disease, and smoking history. We obtained the occurrence of death and major adverse cardiovascular and cerebrovascular events (MACCEs) through outpatient clinics or telephone calls and assessed stent-related complications using ultrasound or computed tomography angiography of the abdominal aorta. All patients were followed up at 1, 6, and 12 months after EVAR and once a year thereafter. Dyslipidemia was defined as a large range of lipid abnormalities or their combination of increased TC ≥ 6.20 mmol/L, LDL-C > 4.13 mmol/L, and triglyceride (TG) levels >2.25 mmol/L or decreased HDL-C < 1.03 mmol (13). We collected blood samples from all patients after fasting for 12 h to detect lipid levels based on the lipid levels of the local population and defined dyslipidemia as the presence of any of the following criteria: (1) Blood TG level of >1.83 mmol/L; (2) Blood TC level of >5.70 mmol/L; (3) Blood LDL-C level >4.0 mmol/L; (4) Blood HDL-C level <0.90 mmol/L, or a combination of these features. Stent-related complications included endoleaks, stent displacement, and stent occlusion. Type I endoleak includes type Ia and type Ib endoleaks, while type II includes transient and persistent endoleaks. MACCEs include newly developed coronary artery disease (CAD), myocardial infarction (MI), and stroke after EVAR. Aneurysm-related death was defined as death within 30 days of EVAR or death associated with stent-related complications.

Stent-related complications were the primary outcome, and secondary outcomes included MACCEs and all-cause mortality.

## Statistical analysis

Statistical analysis was performed using SPSS, version 25, IBM. Continuous variables were represented as mean ± standard deviation (*X* ± SD) and compared using independent sample *t*-tests or Mann–Whitney *U*-test. The categorical variables were expressed as percentages and compared using *χ*^2^ tests. Endpoint events were analyzed using Kaplan–Meier survival curves. Cox regression model was used to evaluate the impact of each predictive factor on outcomes, we selected predictive factors with a univariate analysis *P*-value <0.1 for multivariate analysis to assess the independent effect of each predictor. Two-sided *P* < 0.05 was considered statistically significant.

## Results

### Baseline characteristics

As shown in Table 1, 271 (42.3%) of the 641 patients with AAA had concomitant dyslipidemia. The average age of patients with AAA was 71.06 ± 8.56 years old, with male patients accounting for 82.3%. Patients in the dyslipidemia group had a higher smoking history rate (56.5% vs. 64.6%, *P* = 0.039). There was no statistically significant difference in the rates of hypertension (66.8% vs. 66.1%, *P* = 0.852), chronic obstructive pulmonary disease (18.9% vs. 15.9%, *P* = 0.317), carotid artery disease (4.3% vs. 3.3%, *P* = 0.517), previous stroke (6.5% vs. 6.3%, *P* = 0.913), CAD (17.6% vs. 19.2%, *P* = 0.600), and previous percutaneous coronary intervention (11.6% vs. 12.5%, *P* = 0.722) between the two groups. The prevalence of diabetes (10.0% vs. 15.1%, *P* = 0.050), peripheral arterial disease (17.3% vs. 25.8%, *P* = 0.018), and chronic kidney disease (CKD) (3.0% vs. 6.3%, *P* = 0.043) was higher in the dyslipidemia group than in the non-dyslipidemia group. There was no significant difference in anatomical parameters such as neck diameter (21.22 ± 0.72 vs. 21.16 ± 2.86, *P* = 0.869), neck length (27.91 ± 12.46 vs. 28.78 ± 13.24, *P* = 0.278), and maximum diameter of the AAA between the two groups. The plasma TG (1.09 ± 0.38 vs. 2.24 ± 1.59, *P* < 0.001), TC (4.15 ± 0.79 vs. 4.87 ± 1.50, *P* < 0.001), and LDL-C (2.47 ± 0.66 vs. 3.01 ± 1.21, *P* < 0.001) of patients in the dyslipidemia group were significantly higher than those of non-dyslipidemia group, while HDL-C (1.30 ± 0.32 vs. 1.00 ± 0.32, *P* < 0.001) was significantly lower in the dyslipidemia group.

**Table 1:** Baseline of characteristics.

	Total cases (*n* = 641)	Non-dyslipidemia group (*n* = 370)	Dyslipidemia group (*n* = 271)	*P*-value*
Age, years	71.06 ± 8.56	71.42 ± 8.70	70.56 ± 8.37	0.807
Male	534 (83.3)	304 (82.2)	230 (84.9)	0.364
Smoke history	384 (59.9)	209 (56.5)	175 (64.6)	0.039
Hypertension	426 (66.5)	247 (66.8)	179 (66.1)	0.852
Diabetes mellitus	78 (12.2)	37 (10.0)	41 (15.1)	0.050
COPD	113 (17.6)	70 (18.9)	43 (15.9)	0.317
Carotid artery disease	25 (3.9)	16 (4.3)	9 (3.3)	0.517
Previous stroke	41 (6.4)	24 (6.5)	17 (6.3)	0.913
CAD	117 (18.3)	65 (17.6)	52 (19.2)	0.600
Previous PCI	77 (12.0)	43 (11.6)	34 (12.5)	0.722
PAD	137 (21.4)	67 (17.3)	70 (25.8)	0.018
CKD	28 (4.4)	11 (3.0)	17 (6.3)	0.043
Ruptured AAA	10 (1.6)	6 (1.6)	4 (1.5)	1.000
Neck diameter, mm	21.20 ± 2.78	21.22 ± 0.72	21.16 ± 2.86	0.592
Neck length, mm	28.28 ± 12.79	27.91 ± 12.46	28.78 ± 13.24	0.465
MAD, mm	54.84 ± 13.66	55.16 ± 13.07	54.40 ± 14.45	0.227
TG, mmol/L	1.58 ± 1.21	1.09 ± 0.38	2.24 ± 1.59	<0.001
TC, mmol/L	4.45 ± 1.20	4.15 ± 0.79	4.87 ± 1.50	<0.001
LDL-C, mmol/L	2.70 ± 0.97	2.47 ± 0.66	3.01 ± 1.21	<0.001
HDL-C, mmol/L	1.17 ± 0.35	1.30 ± 0.32	1.00 ± 0.32	<0.001

Data are presented as mean ± standard deviation or percentage (%), *P-*values were computed with Mann–Whitney *U*-test, independent sample *t*-tests or *χ*^2^ tests.

COPD, chronic obstructive pulmonary disease; AAA, abdominal aortic aneurysm; CAD, coronary artery disease; PCI, percutaneous coronary intervention; CKD, chronic kidney disease; MAD, maximal abdominal aortic aneurysm diameter; TG, triglyceride; TC, total cholesterol; HDL-C, high density lipoprotein cholesterol; LDL-C, low density lipoprotein cholesterol.

* *P-*value comparison between two groups.

### Follow-up outcomes

As shown in Table 2, all patients were followed up for more than three years, with an average follow-up time of 63.37 ± 26.49 months. There was no difference in follow-up time between the two groups (63.10 ± 26.89 vs. 63.75 ± 25.98, *P* = 0.760).

**Table 2:** Three-year follow-up outcomes.

	Total cases (*n* = 641)	Non-dyslipidemia group (*n* = 370)	Dyslipidemia group (*n* = 271)	*P*-value*
Duration (months)	63.37 ± 26.49	63.10 ± 26.89	63.75 ± 25.98	0.852
Death	64 (9.98)	38 (10.27)	26 (9.59)	0.778
AAA-associated death	8 (1.25)	5 (1.35)	3 (1.11)	1.000
MACCEs	37 (5.77)	11 (2.97)	26 (9.59)	<0.001
Cardiovascular event	26 (4.06)	10 (2.70)	16 (5.90)	0.042
Stroke	11 (1.72)	1 (0.27)	10 (3.69)	0.001
Stent-related complication	128 (19.97)	82 (22.16)	46 (16.97)	0.105
Type I endoleak	45 (7.02)	34 (9.19)	11 (4.06)	0.012
Type Ia endoleak	15 (2.34)	12 (3.24)	3 (1.11)	0.077
Type Ib endoleak	30 (4.68)	22 (5.95)	8 (2.95)	0.076
Type II endoleak	98 (15.29)	60 (16.22)	38 (14.02)	0.446
Type III endoleak	9 (1.40)	5 (1.35)	4 (1.48)	1.000
Displacement	1 (0.16)	1 (0.27)	0	1.000
Occlusion	8 (1.25)	3 (0.81)	5 (1.85)	0.293

Data are presented as mean ± standard deviation or percentage (%). *P*-values were computed with Mann–Whitney *U*-test or *χ*^2^ tests.

MACCEs, major adverse cardiovascular and cerebrovascular events; MI, myocardial infarction.

* *P*-value comparison between two groups.

### Stent related complications

**Figure 2:**
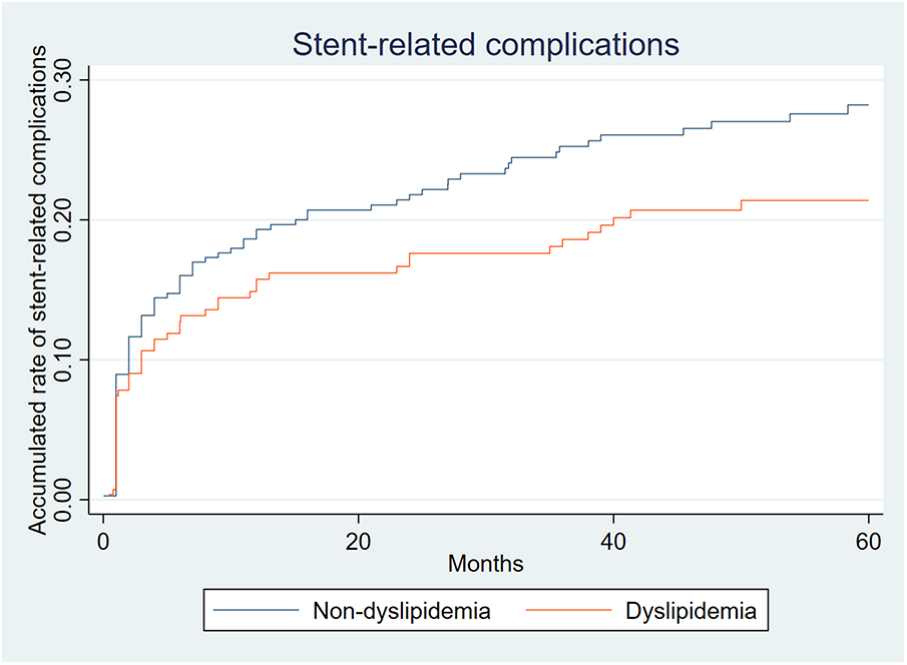
Accumulated rate of stent-related complications.

A total of 128 (19.97%) patients experienced stent-related complications, including 82 (22.16%) in the non-dyslipidemia group and 46 (16.97%) in the dyslipidemia group. Among all complications, the incidence of type II endoleak was the highest (98/641, 15.29%), and its incidence between the two groups was comparable (16.22% vs. 14.02%, *P* = 0.446). This was followed by type Ib endoleak (30/641, 4.68%) with comparable incidence between the two groups (5.95% vs. 2.95%, *P* = 0.076). Only nine type III endoleaks were observed, and there was no difference in the incidence between the two groups (1.35% vs. 1.48%, *P* = 1.00). A total of 45 (7.02%) type I endoleaks were observed, and the incidence of type I endoleaks in the dyslipidemia group was lower than that in the non-dyslipidemia group (9.19% vs. 4.06%, *P* = 0.012). After observing the simultaneous occurrence of various types of endoleaks in some patients, 152 endoleaks were noted. One stent displacement and eight iliac branch occlusions were observed. There was no significant difference in the rate of stent-related complications (22.16% vs. 16.97%, *P* = 0.105) between the two groups, except for type I endoleak. However, as shown in Figure 2, the incidence of stent-related complications in the non-dyslipidemia group was slightly higher than that in the dyslipidemia group.

## Mortality

**Figure 3:**
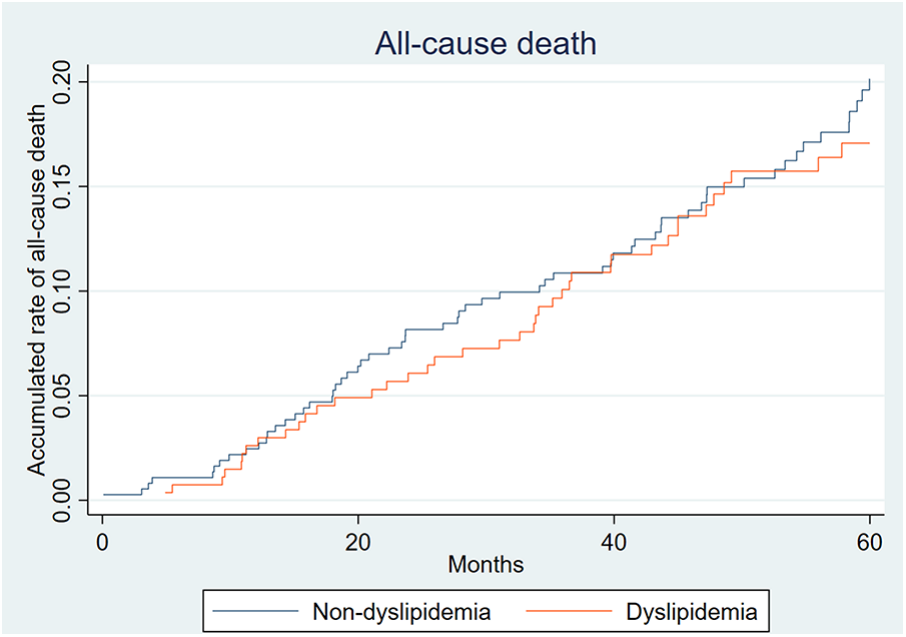
Accumulated rate of all-cause death.

The all-cause mortality rate 3 years after EVAR was 9.98% (64/641). The rate of aneurysm-related death was 1.23% (8/641). From Figure 3, it can be seen that the all-cause mortality rates of the two groups were similar. There was no significant difference in all-cause mortality between the two groups (10.27% vs. 9.59%, *P* = 0.778).

## MACCEs

**Figure 4:**
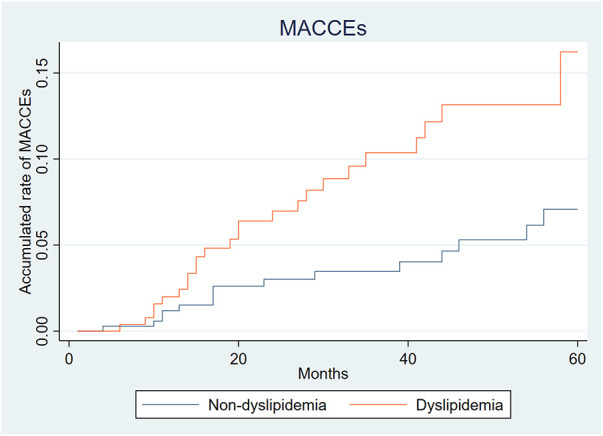
Accumulated rate of major adverse cardiovascular and cerebrovascular events (MACCEs).

A total of 37 (5.77%) MACCEs were observed within 3 years, including 26 cardiovascular events and 11 strokes. The incidence of MACCEs (2.97% vs. 9.59%, *P* < 0.001) in the dyslipidemia group were higher than that in the non-dyslipidemia group. Moreover, as shown in Figure 4, the difference in the incidence of MACCEs between the 2 groups gradually increased within 3 years after EVAR.

### Analysis for follow-up outcomes

**Figure 5:**
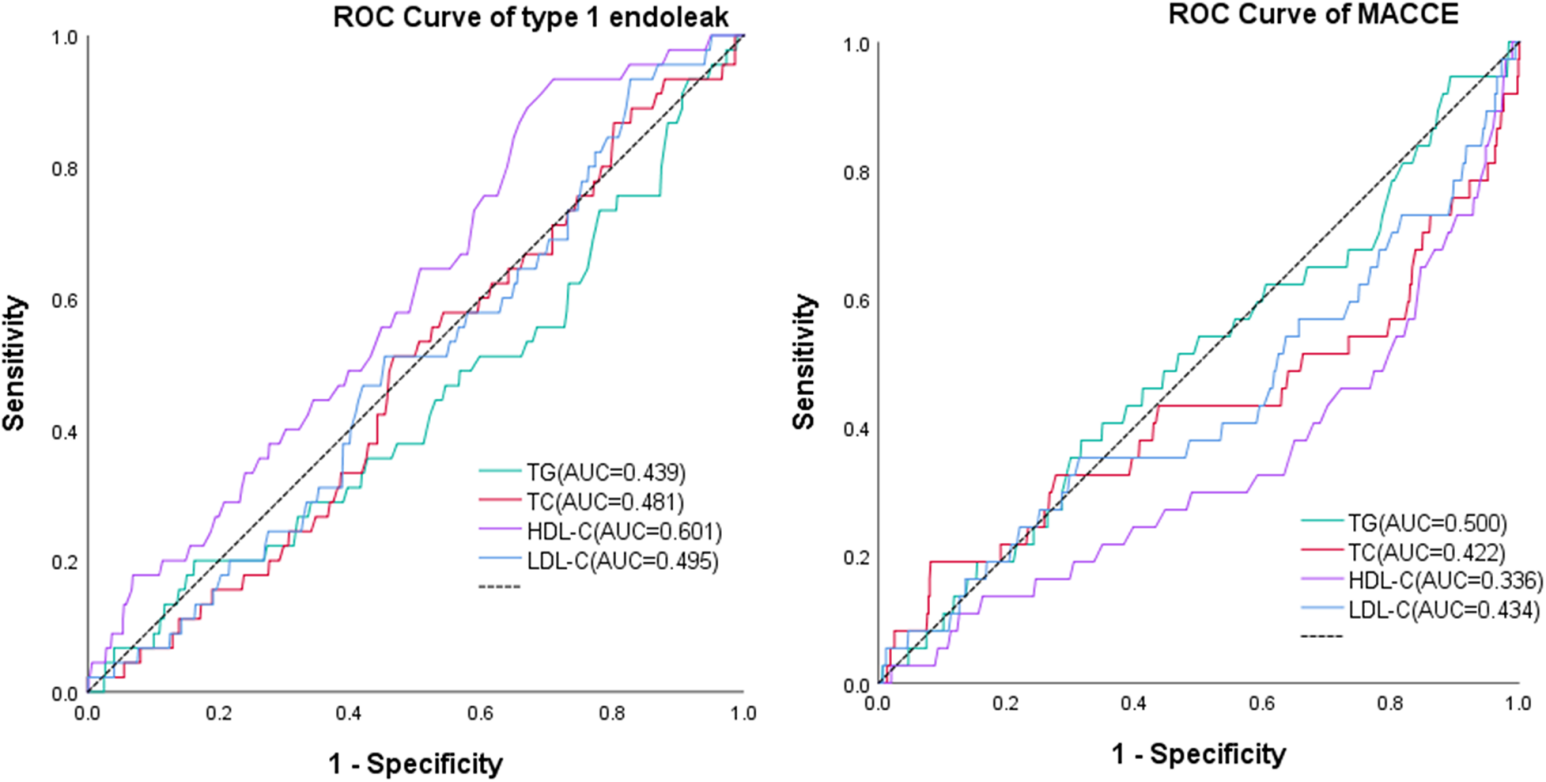
ROC analysis of TG, TC, HDL-C, and LDL-C compared to MACCEs and type 1 endoleak.

As shown in Table 3 and Figure 5, multivariate analysis showed that high level of HDL-C was the protective factor (HR, 0.203, 95% CI, 0.067–0.616, *P* = 0.005) for MACCES, but it was the risk factor for type I endoleak (HR, 2.317, 95% CI, 1.202–4.466, *P* = 0.012), ROC analysis showed that the level of HDL-C can be used to predict Type I endoleak (AUC = 0.601). Smoking history was the protective factor (HR, 0.529, 95% CI, 0.293–0.955, *P* = 0.034) for type I endoleak, the larger MAD was the risk factor (HR, 1.027, 95% CI, 1.009–1.047, *P* = 0.004) for type I endoleak.

**Table 3:** Univariate and multivariate analyses for predictors of follow-up outcomes.

Variable		Univariate analysis		Multivariate analysis	
		HR (95% CI)	*P-*value	HR (95% CI)	*P-*value
MACCE	Diabetes mellitus	1.108 (0.432–2.842)	0.832		
	PAD	1.044 (0.477–2.284)	0.914		
	CKD	2.044 (0.628–6.656)	0.235		
	Smoking history	0.867 (0.452–1.661)	0.666		
	MAD	1.021 (1.000–1.044)	0.053	1.018 (0.996–1.040)	0.114
	TG	1.012 (0.783–1.308)	0.927		
	TC	0.774 (0.569–1.053)	0.102		
	HDL-C	0.181 (0.059–0.556)	0.003	0.203 (0.067–0.616)	0.005
	LDL-C	0.848 (0.589–1.222)	0.376		
Type 1 endoleak	Diabetes mellitus	0.859 (0.339–2.176)	0.748		
	PAD	0.939 (0.452–1.949)	0.866		
	CKD	2.747 (0.983–7.679)	0.054	2.790 (0.979–7.950)	0.055
	Smoking history	0.500 (0.278–0.900)	0.021	0.529 (0.293–0.955)	0.034
	MAD	2.115 (1.179–3.795)	0.012	1.027 (1.009–1.047)	0.004
	TG	0.843 (0.600–1.185)	0.326		
	TC	0.909 (0.704–1.173)	0.463		
	HDL-C	2.170 (1.141–4.126)	0.018	2.317 (1.202–4.466)	0.012
	LDL-C	0.948 (0.697–1.288)	0.732		
Death	Diabetes mellitus	1.037 (0.494–2.174)	0.924		
	PAD	1.510 (0.875–2.603)	0.139		
	CKD	1.661 (0.604–4.571)	0.326		
	Smoking history	1.202 (0.721–2.003)	0.480		
	MAD	1.016 (1.000–1.033)	0.055	1.012 (0.995–1.029)	0.156
	TG	0.734 (0.515–1.046)	0.087	0.838 (0.567–1.240)	0.378
	TC	0.581 (0.457–0.740)	<0.001	0.753 (0.376–1.506)	0.423
	HDL-C	0.520 (0.241–1.124)	0.096	0.690 (0.280–1.700)	0.420
	LDL-C	0.542 (0.395–0.745)	<0.001	0.792 (0.356–1.760)	0.567

*P*-values were computed with Cox regression model.

PAD, peripheral artery disease; CKD, chronic kidney disease; MAD, max aneurysm diameter; TG, triglyceride; TC, total cholesterol; HDL-C, high-density lipoprotein cholesterol; LDL-C, low-density lipoprotein cholesterol.

## Discussion

Dyslipidemia includes a series of lipid abnormalities that may involve elevated TG, TC, and LDL-C levels or a decrease in HDL-C (13). There are regional differences in blood lipid levels, which are related to economic level and dietary structure (14). Our definition of dyslipidemia was based on the distribution of blood lipid levels in the population of our region, which may differ from the blood lipid limit values of other institutions for dyslipidemia. Due to the population distribution characteristics of patients with AAA, dyslipidemia is a common risk factor and comorbidity of AAA (15, 16). A previous study reported an 47.5% incidence of dyslipidemia in patients with AAA, close to 42.3% reported in our study (12). Patients with CKD have a characteristic lipid pattern of hypertriglyceridemia and reduced level of HDL-C (17), which were also often seen in patients with diabetes (18). Smoking is widely recognized as a risk factor for dyslipidemia (19), and dyslipidemia is a risk factor for peripheral artery disease (20). As shown in Table 1, the rates of CKD (3.0% vs. 6.3%, *P* = 0.043), diabetes (10.0% vs.15.1%, *P* = 0.050), smoking history (56.5% vs. 64.6%, *P* = 0.039), and PAD (17.3% vs. 25.8%, *P* = 0.018) in the dyslipidemia group were higher than those in the non-dyslipidemia group. Our study outcomes were consistent with the above viewpoints. Dyslipidemia is an important risk factor for CAD and stroke (13, 21), which is consistent with our study outcome that the dyslipidemia group had a higher rate of MACCEs (2.97% vs. 9.59%, *P* < 0.001), but there was no difference in the incidence of preoperative CAD (17.6% vs. 19.2%, *P* = 0.517), PCI (11.6% vs.12.5%, *P* = 0.722) and stroke (6.5% vs. 6.3%, *P* = 0.913) between the two groups.

Dyslipidemia is more common among people in high-income countries; however, due to dietary structure changes worldwide, its prevalence is increasing, even in low-income countries, particularly in rapidly developing China (14, 22, 23). In the future, the proportion of concomitant dyslipidemia in patients with AAA is predicted to increase. There is still a lack of research on the impact of dyslipidemia on the prognosis of patients with AAA after EVAR. This may be the first study conducted in southwestern China to explore the impact of dyslipidemia on the prognosis of patients with AAA after EVAR. Our study outcomes show that dyslipidemia will not increase the all-cause mortality incidence (10.27% vs. 9.59%, *P* = 0.778) and overall stent-related complications incidence (22.16% vs. 16.97%) in patients with AAA who underwent EVAR, but it will increase the risk of MACCEs (2.97% vs. 9.59%, *P* < 0.001) in patients with AAA who underwent EVAR, specially, it will increase the risk of type I endoleak in patients with AAA who underwent EVAR (9.19% vs. 4.06%, *P* = 0.012), ROC analysis also showed that the level of HDL-C can be used to predict Type I endoleak (AUC = 0.601). Currently, research on the effects of dyslipidemia on the formation of type I endoleak after EVAR is limited, why elevated HDL-C increased the risk of type I endoleak was unclear, there may be a lack of theoretical basis to explain it, this may also be a coincidence limited to statistics, and we need more researches to explore it. The relationship between elevated HDL-C levels and MACCEs risk is still controversial (24, 25), our study outcome suggested that high level of HDL-C was the protective factor (HR, 0.203, 95% CI, 0.067–0.616, *P* = 0.005) for MACCES, the larger MAD was the risk factor (HR, 1.027, 95% CI, 1.009–1.047, *P* = 0.004) for type I endoleak, which was consistent with the outcome of previous study (26). Why patient with smoking hirtory had a lower risk of type I endoleak (HR, 0.529, 95% CI, 0.293–0.955, *P* = 0.034) was unclear, this may be related to patients strictly quitting smoking and staying away from second-hand smoking environments after surgery.

A study pointed out that type II endoleak in patients with AAA after EVAR was associated with dyslipidemia (12); however, the main risk factors for type II endoleak include patent inferior mesenteric artery and patent lumbar artery (27, 28) Whether type II endoleaks can affect the formation of type II endoleaks after EVAR remains to be confirmed. The incidence of type II endoleaks in our study was 15.29%, which was lower than the 19.2% reported in a previous study (29), as shown in Table 2. There was no difference in the incidence of type II endoleaks between the two groups (16.22% vs. 14.02%, *P* = 0.446). In addition, dyslipidemia is closely related to atherosclerosis (30, 31), which also affects the lumbar and inferior mesenteric arteries. It may influence the patency of these arteries and may even reduce the incidence of type II endoleaks. The incidence of type III endoleaks, stent displacement, and iliac branch occlusion was low because of advancements in material technology and surgical techniques. The 2021 Canadian Cardiovascular Society Guidelines state that patients with AAA, dyslipidemia, and clinical atherosclerosis can benefit from statin therapy (32). Each patient with dyslipidemia was advised to take statins after EVAR, even after receiving treatment with statins, some patients' blood lipid levels were not well controlled, which may have been caused by irregular medication use, TG-rich lipoproteins and LDL particles were found to play key roles in the onset and development of cardiovascular disease (33, 34), we can see the incidence of MACCEs after EVAR was still significantly higher in the dyslipidemia group. Furthermore, many patients with dyslipidemia do not make dietary adjustments. For patients with dyslipidemia, more comprehensive management and treatment are needed, and it is imperative to monitor patients in order to prevent the transition to a state of dyslipidemia, with patient compliance playing a pivotal role.

Our study has a few limitations. First, this was a single-center retrospective study with small sample size. Second, due to the interval between postoperative follow-up examinations, records of endoleak occurrence time points may be inaccurate. Third, the completion of statin treatment in patients with dyslipidemia after EVAR has not been effectively monitored. Fourth, many patients do not return to the hospital for follow-up as planned, resulting in many patients being lost during midway visits; thus, postoperative management needs to be strengthened.

## Conclusion

Dyslipidemia does not affect the mortality of patients with AAA who undergo EVAR but may increase the incidence of MACCEs. Dyslipidemia may decrease the incidence of type I endoleaks after EVAR; however, further studies are warranted. We should strengthen the postoperative management of patients with dyslipidemia, prevent the occurrence of MACCEs.

## Data Availability

The raw data supporting the conclusions of this article will be made available by the authors, without undue reservation.

## References

[1] Baman JR Eskandari MK. What is an abdominal aortic aneurysm? JAMA. (2022) 328(22):2280. 10.1001/jama.2022.18638 36511924

[2] Haque K Bhargava P. Abdominal aortic aneurysm. Am Fam Physician. (2022) 106(2):165–72. 35977132

[3] Anagnostakos J Lal BK. Abdominal aortic aneurysms. Prog Cardiovasc Dis. (2021) 65:34–43. 10.1016/j.pcad.2021.03.009 33831398

[4] Gao JP Guo W. Mechanisms of abdominal aortic aneurysm progression: a review. Vasc Med. (2022) 27(1):88–96. 10.1177/1358863X211021170 34278882

[5] Schanzer A Oderich GS. Management of abdominal aortic aneurysms. N Engl J Med. (2021) 385(18):1690–8. 10.1056/NEJMcp2108504 34706173

[6] Chaikof EL Dalman RL Eskandari MK Jackson BM Lee WA Mansour MA The society for vascular surgery practice guidelines on the care of patients with an abdominal aortic aneurysm. J Vasc Surg. (2018) 67(1):2–77.e2. 10.1016/j.jvs.2017.10.044 29268916

[7] Li C de Guerre L Dansey K Lu J Patel PB Yao M The impact of completion and follow-up endoleaks on survival, reintervention, and rupture. J Vasc Surg. (2023) 77(6):1676–84. 10.1016/j.jvs.2023.02.009 36841312 PMC10213115

[8] Akmal MM Pabittei DR Prapassaro T Suhartono R Moll FL van Herwaarden JA. A systematic review of the current status of interventions for type II endoleak after EVAR for abdominal aortic aneurysms. Int J Surg. (2021) 95:106138. 10.1016/j.ijsu.2021.106138 34637951

[9] Weng LC Roetker NS Lutsey PL Alonso A Guan W Pankow JS Evaluation of the relationship between plasma lipids and abdominal aortic aneurysm: a Mendelian randomization study. PLoS One. (2018) 13(4):e0195719. 10.1371/journal.pone.0195719 29649275 PMC5896990

[10] Nastasi DR Norman R Moxon JV Quigley F Velu R Jenkins J The potential benefits and costs of an intensified approach to low density lipoprotein cholesterol lowering in people with abdominal aortic aneurysm. Eur J Vasc Endovasc Surg. (2021) 62(4):643–50. 10.1016/j.ejvs.2021.06.031 34507892

[11] Martínez-López D Cedó L Metso J Burillo E García-León A Canyelles M Impaired HDL (high-density lipoprotein)-mediated macrophage cholesterol efflux in patients with abdominal aortic aneurysm-brief report. Arterioscler Thromb Vasc Biol. (2018) 38(11):2750–4. 10.1161/ATVBAHA.118.311704 30354236

[12] Hall MR Protack CD Assi R Williams WT Wong DJ Lu D Metabolic syndrome is associated with type II endoleak after endovascular abdominal aortic aneurysm repair. J Vasc Surg. (2014) 59(4):938–43. 10.1016/j.jvs.2013.10.081 24360238 PMC3966942

[13] Kopin L Lowenstein C. Dyslipidemia. Ann Intern Med. (2017) 167(11):81–96. 10.7326/AITC201712050 29204622

[14] Pirillo A Casula M Olmastroni E Norata GD Catapano AL. Global epidemiology of dyslipidaemias. Nat Rev Cardiol. (2021) 18(10):689–700. 10.1038/s41569-021-00541-4 33833450

[15] Golledge J. Abdominal aortic aneurysm: update on pathogenesis and medical treatments. Nat Rev Cardiol. (2019) 16(4):225–42. 10.1038/s41569-018-0114-9 30443031

[16] Sakalihasan N Michel JB Katsargyris A Kuivaniemi H Defraigne JO Nchimi A Abdominal aortic aneurysms. Nat Rev Dis Primers. (2018) 4(1):34. 10.1038/s41572-018-0030-7 30337540

[17] Ferro CJ Mark PB Kanbay M Sarafidis P Heine GH Rossignol P Lipid management in patients with chronic kidney disease. Nat Rev Nephrol. (2018) 14(12):727–49. 10.1038/s41581-018-0072-9 30361677

[18] Goldberg RB. Dyslipidemia in diabetes: when and how to treat? Endocrinol Metab Clin North Am. (2022) 51(3):603–24. 10.1016/j.ecl.2022.02.011 35963631

[19] Jeong W. Association between dual smoking and dyslipidemia in South Korean adults. PLoS One. (2022) 17(7):e0270577. 10.1371/journal.pone.0270577 35802704 PMC9269882

[20] Berger JS Hochman J Lobach I Adelman MA Riles TS Rockman CB. Modifiable risk factor burden and the prevalence of peripheral artery disease in different vascular territories. J Vasc Surg. (2013) 58(3):673–81.1. 10.1016/j.jvs.2013.01.053 23642926

[21] Arvanitis M Lowenstein CJ. Dyslipidemia. Ann Intern Med. (2023) 176(6):81–96. 10.7326/AITC202306200 37307585

[22] Opoku S Gan Y Yobo EA Tenkorang-Twum D Yue W Wang Z Awareness, treatment, control, and determinants of dyslipidemia among adults in China. Sci Rep. (2021) 11(1):10056. 10.1038/s41598-021-89401-2 33980884 PMC8115030

[23] Xing L Jing L Tian Y Yan H Zhang B Sun Q Epidemiology of dyslipidemia and associated cardiovascular risk factors in northeast China: a cross-sectional study. Nutr Metab Cardiovasc Dis. (2020) 30(12):2262–70. 10.1016/j.numecd.2020.07.032 32988725

[24] Bowe B Xie Y Xian H Balasubramanian S Zayed MA Al-Aly Z. High density lipoprotein cholesterol and the risk of all-cause mortality among U.S. veterans. Clin J Am Soc Nephrol. (2016) 11(10):1784–93. 10.2215/CJN.00730116 27515591 PMC5053782

[25] Kaur M Ahuja KR Khubber S Zhou L Verma BR Meenakshisundaram C Effect of high-density lipoprotein cholesterol levels on overall survival and major adverse cardiovascular and cerebrovascular events. Am J Cardiol. (2021) 146:8–14. 10.1016/j.amjcard.2021.01.014 33535058

[26] Zuccon G D'Oria M Gonçalves FB Fernandez-Prendes C Mani K Caldeira D Incidence, risk factors, and prognostic impact of type Ib endoleak following endovascular repair for abdominal aortic aneurysm: scoping review. Eur J Vasc Endovasc Surg. (2023) 66(3):352–61. 10.1016/j.ejvs.2023.06.017 37356703

[27] Müller-Wille R Güntner O Zeman F Dollinger M Hälg C Beyer LP The influence of preoperative aneurysmal thrombus quantity and distribution on the development of type II endoleaks with aneurysm sac enlargement after EVAR of AAA. Cardiovasc Intervent Radiol. (2016) 39(8):1099–109. 10.1007/s00270-016-1386-2 27307180

[28] Lo RC Buck DB Herrmann J Hamdan AD Wyers M Patel VI Risk factors and consequences of persistent type II endoleaks. J Vasc Surg. (2016) 63(4):895–901. 10.1016/j.jvs.2015.10.088 26796291 PMC4808613

[29] Mulay S Geraedts ACM Koelemay MJW Balm R. Type 2 endoleak with or without intervention and survival after endovascular aneurysm repair. Eur J Vasc Endovasc Surg. (2021) 61(5):779–86. 10.1016/j.ejvs.2021.01.017 33632609

[30] Sandesara PB Virani SS Fazio S Shapiro MD. The forgotten lipids: triglycerides, remnant cholesterol, and atherosclerotic cardiovascular disease risk. Endocr Rev. (2019) 40(2):537–57. 10.1210/er.2018-00184 30312399 PMC6416708

[31] Hurtubise J McLellan K Durr K Onasanya O Nwabuko D Ndisang JF. The different facets of dyslipidemia and hypertension in atherosclerosis. Curr Atheroscler Rep. (2016) 18(12):82. 10.1007/s11883-016-0632-z 27822682

[32] Pearson GJ Thanassoulis G Anderson TJ Barry AR Couture P Dayan N 2021 Canadian cardiovascular society guidelines for the management of dyslipidemia for the prevention of cardiovascular disease in adults. Can J Cardiol. (2021) 37(8):1129–50. 10.1016/j.cjca.2021.03.016 33781847

[33] Zhang BH Yin F Qiao YN Guo SD. Triglyceride and triglyceride-rich lipoproteins in atherosclerosis. Front Mol Biosci. (2022) 9:909151. 10.3389/fmolb.2022.909151 35693558 PMC9174947

[34] Qiao YN Zou YL Guo SD. Low-density lipoprotein particles in atherosclerosis. Front Physiol. (2022) 13:931931. 10.3389/fphys.2022.931931 36111155 PMC9468243

